# Individual and Interactive Effects of Housing and Neighborhood Quality on Mental Health in Hong Kong: A Retrospective Cohort Study

**DOI:** 10.1007/s11524-024-00869-5

**Published:** 2024-05-08

**Authors:** Corine Sau Man Wong, Wai Chi Chan, Natalie Wing Tung Chu, Wing Yan Law, Harriet Wing Yu Tang, Ting Yat Wong, Eric Yu Hai Chen, Linda Chiu Wa Lam

**Affiliations:** 1 School of Public Health, LKS Faculty of Medicine, The University of Hong Kong, Hong Kong SAR, China; 2 Department of Psychiatry, School of Clinical Medicine, LKS Faculty of Medicine, The University of Hong Kong, Hong Kong SAR, China; 3 Department of Psychiatry, Faculty of Medicine, The Chinese University of Hong Kong, Hong Kong SAR, China; 4 Department of Psychology, The Education University of Hong Kong, Hong Kong SAR, China; 5 Lee Kong Chian School of Medicine, Nanyang Technological University, Singapore, Singapore; 6 Department of Psychosis, Institute of Mental Health, Singapore, Singapore; 7 State Key Laboratory of Brain and Cognitive Sciences, The University of Hong Kong, Hong Kong SAR, China

**Keywords:** Housing, Neighborhood, Mental health, Urban environment

## Abstract

Existing literature has widely explored the individual roles of housing and neighborhood quality, and there is limited research examining their interactive effects on mental health. This 3-year cohort study utilized a longitudinal design to investigate the individual and interactive effects of housing and neighborhood quality on mental health among 962 community-dwelling adults in Hong Kong. Participants were asked to rate their residential qualities over the 3-year period. Mental health outcomes, including levels of psychological distress and common mental disorders (CMD), were assessed using the Revised Clinical Interview Schedule (CIS-R). Logistic regression and generalized linear models were used to examine the association between housing and neighborhood quality and CMD/psychological distress, adjusting for sociodemographic and residential characteristics and baseline mental disorders. Housing quality was associated with the 3-year CMD (adjusted OR 0.95; 95% CI 0.91 to 0.98). Likewise, neighborhood quality was associated with CMD over 3 years (adjusted OR 0.92; 95% CI 0.87 to 0.96). In a separate model including both quality measures, the effect of housing quality on CMD was attenuated, whereas the neighborhood impact remained significant (adjusted OR 0.92; 95% CI 0.87 to 0.98). Generalized linear models indicated that for participants residing in substandard housing, those with high neighborhood quality had lower CIS-R scores at follow-up compared to those with low neighborhood quality (*p* = 0.041). Better neighborhood quality alleviated the detrimental effects of poor housing quality on mental health. Planning for an enhanced neighborhood would improve population mental health in an urban environment.

**Supplementary Information:**

The online version contains supplementary material available at 10.1007/s11524-024-00869-5.

## Introduction

Urban living environment is the key determinant of population health [1]. Evidence has shown that people who live in favored facilities experience better physical health, fewer chronic illnesses, and lower mortality rates [2–4]. In contrast, residing in an uncomfortable environment induces both short- and long-term stress, which causes permanent harm to health [5].

The significance of the living environment, encompassing both housing and neighborhood quality, has been recognized as a pivotal factor influencing mental health [6]. Poor housing quality, such as overcrowding, inadequate ventilation and dampness, have been associated with increased risks of mental disorders [7]. Neighborhood characteristics, such as safety, noise level, access to amenities and green space, have been linked to mental well-being [8]. Many of these studies have highlighted the potential impact of housing and neighborhood characteristics on mental health. However, due to the cross-sectional nature of the studies, establishing causal relationships between the living environment and mental health has been challenging [9].

While the individual roles of housing and neighborhood quality have been extensively studied [5], there is a paucity of research investigating their interactive effects on mental health. It has been hypothesized that neighborhoods lacking public facilities, secure environments and decent housing may impose stress on residents and increase their risks of depression [10]. In contrast, high neighborhood quality may mitigate the adverse mental health impact of substandard housing [11]. For instance, a supportive neighborhood environment characterized by high social cohesion and resources may buffer the detrimental effects of poor housing conditions, providing residents with a sense of belonging and social support [10]. Conversely, residing in a disadvantaged neighborhood with limited resources and social fragmentation may exacerbate the negative impact of substandard housing on mental health [11]. Studies on the synergistic effects of housing and neighborhood quality could offer insight into how the living environment influences mental health.

This study aimed to replicate and extend the Jones-Rounds et al. study [11] in filling the gap by examining both the individual and interactive effects of housing and neighborhood quality on mental health in a sample of community-dwelling Chinese adults in Hong Kong, a densely populated city situated in Southeastern China with over 7.5 million people. As a prominent urban city, Hong Kong has a diverse population comprising different ethnicities, socioeconomic backgrounds, and cultural norms. These various demographic groups are likely to have differing perceptions of housing and neighborhood quality, which can further influence their mental health outcomes. Understanding these differences and their impact on mental health is crucial for developing targeted interventions and policies to address the unique challenges faced by different population groups in Hong Kong.

Using a population-based cohort, we aimed to investigate how individuals from diverse backgrounds (e.g., education and income level) and residential characteristics (e.g., housing type and size) perceive their living environment, as well as to examine relationships and explore potential causal pathways between living environment and mental health. We hypothesized that the quality of housing and neighborhood will be positively associated with mental health (in particular, lower common mental disorders and psychological distress) over time. In addition, the housing and neighborhood quality were hypothesized to have interactive effects on mental health. Our study aimed to provide a comprehensive understanding of how these two critical environmental factors interplay to shape mental health. The findings would potentially inform and develop policies targeting housing and neighborhood environments to improve mental health in urban populations.

## Methods

### Study Population

Participants in this retrospective cohort study were recruited from the 3-year longitudinal follow-up study of the Hong Kong Mental Morbidity Survey (HKMMS). The HKMMS consists of a representative community-dwelling sample of 5719 Chinese adults between 16 and 75 years recruited from November 2010 to May 2013. A multistage stratified cluster sampling design was adopted, in which one adult from each household was randomly selected for participation. The details of the study method and the main findings have been reported elsewhere [12, 13]. To examine the longitudinal outcomes of mental health, a follow-up study was carried out 3 years after the baseline study. In this study, a subsample of HKMMS participants who had completed the baseline assessment was invited to participate in a 45-min face-to-face interview. To comprehensively assess the effects of the perceived environmental qualities over the general population, 750 participants with common mental disorder (CMD) and 1250 participants without CMD at baseline were invited. Participants with severe mental disorders such as psychotic disorders or dementia were excluded, given the potential cognitive impairment and difficulty in assessment comprehension. Written informed consent was obtained from all participants. Ethical approval was obtained from the Institutional Review Board of the University of Hong Kong/Hospital Authority Hong Kong West Cluster (Reference No. UW15-304).

### Measures

#### Quality of Living Environment

The primary study exposure of interest was the quality of the living environment. At the follow-up interview, participants were asked to respond to 10 items on housing quality (lighting, air quality, hygiene, serenity, spacing, kitchen facilities, toilet facilities, furnishings, architecture and design, and structure safety) and 10 items on neighborhood quality (lighting, air quality, hygiene, serenity, transportation, shopping, catering, medical facilities, park and recreation, and security) over the 3-year interval. Each item ranged from 1 (strongly dissatisfied) to 5 (strongly satisfied) on a 5-point Likert scale with reference to the perceived environmental quality indices [14]. A higher score represented a higher level of perceived quality of the living environment. We adopted subjective evaluations of participants’ living environment because perception of environmental characteristics has been found to be an essential factor of mental health [15, 16]. To mitigate the potential influence of recall bias, we provided participants with clear instructions and utilized standardized scales to assess the quality of housing and neighborhood throughout the follow-up period. The scale achieved good internal consistency for both subscales of housing quality (Cronbach’s alpha = 0.91) and neighborhood quality (Cronbach’s alpha = 0.87), as well as good 1-week test-retest reliability (Correlation coefficient = 0.89).

#### Psychological Distress and CMD

The study outcomes were defined by the level of psychological distress and CMD at the 3-year follow-up. Level of psychological distress was assessed using the Chinese version of the Revised Clinical Interview Schedule (CIS-R) [17]. It measures 14 non-psychotic symptoms, and the score for each section ranges from 0 to 4 (except for the maximum score of 5 in the section of depressive ideas). A higher score reflects a higher severity of psychological distress. The CIS-R was developed from the Clinical Interview Schedule and has been widely used as a structured diagnostic instrument for the assessment of CMD [18]. A cut-off score of ≥12 was defined as diagnostic for the presence of CMD. The Chinese version of the CIS-R was reported to have excellent validity and reliability with satisfactory sensitivity (69.3%) and specificity (92.7%) in detecting diagnosable CMD [17]. Level of psychological distress (CIS-R score) and CMD (CIS-R score of <12 or ≥12) were collected at both baseline (as confounders) and follow-up interviews.

#### Sociodemographic and Residential Characteristics

Sociodemographic and residential characteristics were measured as covariates at baseline, including age, sex, education level, marital status, employment status and household income. Information regarding residence, including the living district, year of residency, housing type (public or private housing), property age, housing size and housing tenure status (owner or renter), was collected with a standardized data form.

### Statistical Analysis

The data analysis involved four steps: (1) descriptive statistics and group comparisons, (2) unadjusted and adjusted logistic regression models, (3) adjusted logistic regression models with interaction terms, and (4) generalized linear models. First, the normality of all variables was checked with the Shapiro-Wilk test. Due to a non-parametric structure, data regarding sociodemographic and residential characteristics were analyzed using the Mann-Whitney *U* and Kruskal-Wallis tests. Pairwise tests with Bonferroni adjustment for multiple comparisons were carried out for any significance detected in the Kruskal-Wallis test. Second, univariate logistic regression analyses were conducted to locate significant variables (*p* < 0.05), which were further processed by stepwise logistic regression analyses to examine their associations with CMD at follow-up. The models were also adjusted for potential confounding factors such as sociodemographic and residential characteristics (age, sex, education level, and household income) and baseline CMD. Third, an interaction term of housing quality and neighborhood quality was added in a separate model to investigate any interaction effects on outcomes. Adjusted odds ratios (aORs) were presented with a 95% confidence interval (CI). The proportion of variance explained by the model was estimated by Nagelkerke pseudo *R*^2^ and the model fit was assessed by the Hosmer-Lemeshow goodness-of-fit test. Lastly, the housing and the neighborhood quality were each split into two groups according to the median scores. The split groups were grouped into four combinations: (1) low housing and low neighborhood quality, (2) low housing and high neighborhood quality, (3) high housing and low neighborhood quality, and (4) high housing and high neighborhood quality. Four generalized linear models on split groups according to perceived housing and environmental quality were fitted with the follow-up CIS-R score as the dependent variable and the group factors as the independent variable, adjusting for confounding factors. Statistical analyses were conducted with the STATA Version 15 (StataCorp, College Station, TX, USA).

## Results

### Participant Characteristics

The study was conducted from November 2014 to November 2016 with a mean duration of 37.1 months (SD 4.0 months; range 32 to 42 months) between the baseline and follow-up interviews. The overall response rate was 51.1% (1005 participants recruited out of 1965 invitations). No significant group difference was found in comparing the characteristics (age, sex, education level and household income) of responders and non-responders (*p* > 0.05). The current study excluded 43 (4.3%) participants who moved to another residence address over the 3-year follow-up period, resulting in a final study population of 962 community-dwelling adults. Table 1 summarizes the sociodemographic and residential characteristics of the participants. The mean age at baseline was 47.5 years (SD 15.2 years), and middle-aged (46 to 60 years) participants were the largest group (33%). Regarding socioeconomic status (SES), 63.8% of the participants had monthly household incomes below Hong Kong Dollar 25,000. About 46.6% resided in public housing and 53.4% in private properties. The median duration of residence was 15 years (range 3 to 51 years), and the median housing size was 450 square feet (range 65 to 2500 square feet).

**Table 1:** Sociodemographic and residential characteristics of the participants (*N* = 962)

	*n* (%)
Baseline age	
16–30	150 (15.6)
31–45	270 (28.1)
46–60	317 (33.0)
61–75	225 (23.4)
Sex	
Male	288 (29.9)
Female	674 (70.1)
Education level	
No schooling/primary	184 (19.1)
Lower secondary	178 (18.5)
Upper secondary	407 (42.3)
Post-secondary	193 (20.1)
Marital status	
Married/cohabit	532 (55.3)
Never married	237 (24.6)
Divorced/separated/widowed	193 (20.1)
Employment status	
Employed	518 (53.8)
Retired	187 (19.4)
Housewife	120 (12.5)
Student	50 (5.2)
Unemployed/not working	87 (9.0)
Monthly household income (HKD)	
<15,000	408 (45.2)
15,000–24,999	168 (18.6)
25,000–39,999	143 (15.9)
40,000–59,999	93 (10.3)
≥60,000	90 (10.0)
District of residence	
Hong Kong Island	134 (13.9)
Kowloon	319 (33.2)
New Territories and Islands	509 (53.0)
Housing type	
Public housing	448 (46.6)
Private housing	514 (53.4)
Housing size (square feet, in quintile)	
≤20%	225 (23.6)
21–40%	218 (22.9)
41–60%	177 (18.6)
61–80%	158 (16.6)
>80%	176 (18.4)
Year of residence	
3–5	124 (13.5)
6–10	198 (21.5)
11–15	174 (18.9)
16–20	156 (17.0)
21–25	114 (12.4)
26–30	95 (10.3)
>30	58 (6.3)
Housing tenure status	
Owner	326 (45.9)
Renter	385 (54.1)
Year of property age	
3–10	42 (4.4)
11–20	280 (29.4)
21–30	310 (32.5)
31–40	207 (21.7)
>40	114 (12.0)

*HKD* Hong Kong Dollar

### Individual Effects of Housing and Neighborhood Quality on Mental Health

Overall, participants who were married (as compared to never married), retired or housewives (as compared to employed), had higher educational level (post-secondary) and higher household income (Hong Kong Dollar 60,000 or above per month) perceived higher housing and neighborhood qualities. Better living quality was reported by participants residing in private housing and tenured housing. Those living in larger-sized housing also reported better housing conditions (Supplementary Table 1 and Table 2). Perceived residential quality was associated with CMD at the 3-year follow-up (Housing quality: aOR 0.95; 95% CI 0.91 to 0.98; *p* = 0.007; Neighborhood quality: aOR 0.92; 95% CI 0.87 to 0.96; *p* < 0.001) after adjusting for confounding variables. The Hosmer-Lemeshow goodness of fit test indicated no evidence of a poor fit (Housing quality: *χ*^2^ = 2.77, df = 8; *p* = 0.948; Neighborhood quality: *χ*^2^ = 7.99, df = 8; *p* = 0.434). The model of housing quality accounted for 39% of the variance (Supplementary Table 3), while the neighborhood quality model explained 40% of the variance (Table 2).

**Table 2:** Association between sociodemographic and residential characteristics, neighborhood quality and CMD at follow-up (*N* = 962)^a^

	B	SE	Wald statistic	aOR (95% CI)
**Step 1**				
Sex (female)	0.19	0.27	0.50	1.21 (0.71–2.08)
Baseline age				
16–30	Reference			
31–45	0.12	0.44	0.07	1.12 (0.47–2.68)
46–60	−0.21	0.46	0.20	0.81 (0.33–2.00)
61–75	−0.67	0.62	1.17	0.51 (0.15–1.72)
Marital status				
Married/cohabit	Reference			
Never married	−0.13	0.36	0.13	0.88 (0.44–1.78)
Divorced/separated/widowed	0.01	0.31	0.00	1.01 (0.55–1.85)
Education level				
No schooling/primary	Reference			
Lower secondary	0.76	0.38	4.10	2.15 (1.03–4.50)^*^
Upper secondary	−0.32	0.38	0.73	0.73 (0.35–1.52)
Post-secondary	−0.38	0.55	0.48	0.68 (0.23–2.01)
Monthly household income (HKD)				
<15,000	Reference			
15,000–24,999	−0.50	0.35	2.10	0.61 (0.31–1.19)
25,000–39,999	−0.72	0.42	2.97	0.49 (0.22–1.10)
40,000–59,999	−0.46	0.49	0.87	0.63 (0.24–1.65)
≥60,000	−0.32	0.54	0.35	0.73 (0.25–2.10)
Employment status				
Employed	Reference			
Retired	−0.67	0.50	1.79	0.51 (0.19–1.37)
Housewife	0.19	0.35	0.29	1.21 (0.60–2.42)
Student	−1.14	0.74	2.35	0.32 (0.08–1.37)
Unemployed/not working	0.66	0.39	2.82	1.94 (0.90–4.18)
**Step 2**				
Public housing	−0.81	0.40	4.08	0.45 (0.20–0.98)^*^
Tenured housing	−1.03	0.37	7.79	0.36 (0.17–0.74)^**^
Year of property age				
3–10	Reference			
11–20	0.57	0.62	0.83	1.76 (0.52–5.93)
21–30	0.99	0.62	2.52	2.68 (0.79–9.08)
31–40	0.72	0.64	1.25	2.05 (0.58–7.17)
>40	0.23	0.71	0.11	1.26 (0.31–5.05)
**Step 3**				
CMD at baseline	2.31	0.24	89.34	10.08 (6.24–16.27)^***^
**Step 4**				
Quality of neighborhood	−0.09	0.02	14.54	0.92 (0.87–0.96)^***^

*B* coefficient on the variable, *CI* confidence interval, *CMD* common mental disorder, *HKD* Hong Kong Dollar, *aOR* adjusted odds ratio, *SE* standard error

^a^Final stepwise logistic regression model: *χ*^2^202.28, df = 25, *p* < 0.001; Nagelkerke *R*^2^ = 0.404

^***^p < 0.001; ^**^p < 0.01; ^*^p < 0.05

### Interactive Effects of Housing and Neighborhood Quality on Mental Health

Due to the multicollinearity between the perceived quality of housing and neighborhood (Supplementary Table 4), we retested the model by including both factors. The neighborhood quality was associated with CMD at follow-up (aOR 0.92; 95% CI 0.87 to 0.98; *p* = 0.005), and the housing effect on CMD was insignificant (aOR 0.99; 95% CI 0.95 to 1.04; *p* = 0.733) (Table 3). In a separate model with the interaction term added, both housing and neighborhood effects were attenuated (*p* > 0.05), and the interaction term was insignificant (aOR 1.00; 95% CI 0.99 to 1.00; *p* = 0.656) (Supplementary Table 5).

**Table 3:** Association between sociodemographic and residential variables, housing and neighborhood quality and CMD at follow-up (*N* = 962)^a,b^

	B	SE	Wald statistic	aOR (95% CI)
**Step 1**				
Sex, Baseline age, Marital status, Education level, Monthly household income, Employment status				
**Step 2**				
Public housing, Tenured housing, Year of property age				
**Step 3**				
Baseline CMD				
**Step 4**				
Quality of housing	−0.01	0.03	0.12	0.99 (0.95–1.04)
Quality of neighborhood	−0.08	0.03	8.04	0.92 (0.87–0.98)^**^

*B* coefficient on the variable, *CI* confidence interval, *CMD* common mental disorder, *aOR* adjusted odds ratio, *SE* standard error

^a^Final stepwise logistic regression model: *χ*^2^ = 202.39, df = 26, *p* < 0.001; Nagelkerke *R*^2^ = 0.404

^b^Housing and neighborhood quality are the variables of interest, details of Step 1–3 are not shown

^***^p < 0.001; ^**^p < 0.01; ^*^p < 0.05

**Fig. 1:**
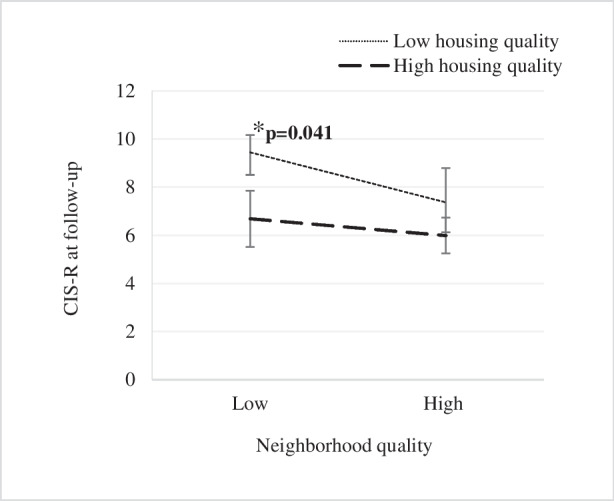
CIS-R score at follow-up associated with low/high neighborhood quality. CIS-R, Revised Clinical Interview Schedule

**Fig. 2:**
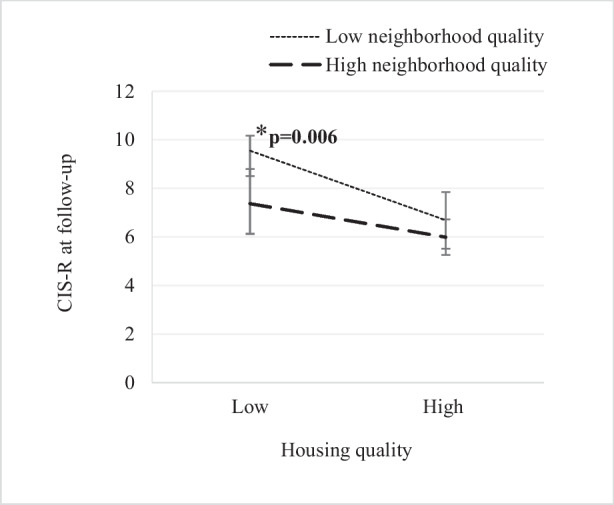
CIS-R score at follow-up associated with low/high housing quality. CIS-R, Revised Clinical Interview Schedule

Figures 1 and 2 show the mean CIS-R score at follow-up according to the qualities of the living environment. Generalized linear models revealed that participants who reported low housing quality, those with a high neighborhood quality had lower CIS-R scores at follow-up than those with a low neighborhood quality (7.47 ± 7.11 versus 9.34 ± 8.24; *p* = 0.041). However, for participants who perceived high housing quality, the CIS-R score did not differ, regardless of the level of neighborhood quality (*p* = 0.333) (Fig. 1). On the other hand, among participants who reported low neighborhood quality, those who perceived high housing quality exhibited a lower CIS-R score at follow-up compared to those who perceived low housing quality (6.69 ± 6.09 versus 9.34 ± 8.24; *p* = 0.006). However, the CIS-R score did not differ between individuals residing in low and high housing quality given high neighborhood quality (*p* = 0.081) (Fig. 2).

## Discussion

To our knowledge, this is the first study examining the longitudinal effects of the quality of housing and neighborhood on mental health in Hong Kong. Our findings demonstrated that living environments have a significant impact on residents’ mental health over time. Higher housing and neighborhood quality lowered the risk of CMD in 3 years after controlling for sociodemographic, dwelling factors and baseline mental disorders. These observations are in line with previous literature that negative perceptions of one’s living environment are associated with an increased risk of mental health problems and vice versa [6, 19, 20].

Consistent with previous studies [21, 22], we found that participants with higher levels of education and household income generally perceived better quality of housing and neighborhood. High-SES individuals had more knowledge and resources to select a dwelling that closely suited their needs, resulting in greater housing and neighborhood satisfaction. Our findings also showed that homemakers, retirees and older adults had higher satisfaction with the quality of their living environment. These individuals spend more time at home or have lived in their homes for a more extended period, which may lead to greater attachment to their residence due to social ties or place identification [15, 23]. Besides, residential characteristics such as homeownership, housing type and housing size contribute to one’s perception of the living environment. Ownership of one’s dwelling has been reported as one of the most substantial factors associated with residential satisfaction [21, 22]. Correspondingly, individuals who own their homes tend to have a more positive perception of their homes and environment, possibly because homeownership allows more freedom in the design and use of residence and that fosters a pronounced sense of autonomy, security and personal identity [23]. Understanding the diverse perspectives of individuals from various backgrounds in relation to housing and neighborhood quality is essential for promoting equitable mental health outcomes. By recognizing these differences, policymakers can develop targeted strategies that address the needs of each population group, leading to effective policies on housing and neighborhood that foster mental health and reduce disparities among different groups.

Although we did not find an interaction effect of housing and neighborhood quality on CMD, our data fit with Jones-Rounds et al. study [11] that housing and neighborhood quality did have potential interactive effects on mental health. Our study revealed that when both housing and neighborhood quality were considered in the model, the impact of housing quality on CMD was attenuated while the neighborhood effect remained significant. Moreover, we found that living in a better neighborhood could help protect against the negative effects of poor housing on psychological distress. These findings highlight the prominent psychological benefits associated with a favorable neighborhood environment. While individuals residing in mediocre housing with uncontrollable factors (e.g., structural defects) possibly experience a sense of learned helplessness that induces psychological distress, they may mitigate the negative impact of housing on their mental well-being by enhancing the psychosocial environment within their neighborhood.

Living in a good neighborhood significantly impacts our mental health for various reasons. Previous evidence has shown that social connectedness in the neighborhood (e.g., engaging in social networking and participating in community activities) is associated with positive mental health outcomes, including enhanced resilience, increased well-being, reduced mood symptoms and decreased vulnerability to future depression [24–26]. Stronger neighborhood cohesion and identification also strengthen the sense of control and buffer against the stressful effects of substandard housing conditions, potentially preventing mental health issues [27, 28]. In addition, a pleasant neighborhood provides residents the opportunity to engage in affordable leisure-time physical activities (e.g., jogging and cycling) [29] and hence lower the risk of depression and anxiety (i.e., a dose-response relationship between physical activity and mental health) [30]. Furthermore, a growing body of evidence reveals that convenient access to public services, amenities, green spaces, and recreational areas can greatly improve quality of life and reduce psychological distress [31]. In sum, an optimal neighborhood environment facilitates social interaction and promotes physical activity; this, in turn, enhances individuals’ resilience in coping with disadvantaged backgrounds and challenging environmental conditions [32, 33].

This study has several strengths. First, we employed a retrospective longitudinal design, which enabled the examination of how individuals’ perception of the living environment was related to mental health outcomes over time. It has overcome the limitations of prior cross-sectional studies. Second, our study sought to establish causal associations by incorporating self-reported environmental attributes and a fully structured interviewer-administered diagnostic measurement, which provided a valid and reliable estimate of CMD in Hong Kong [17]. We could identify not only the links between living environment and psychological distress but also its rarely-explored relationship with mental disorders [8]. Third, using multivariate analyses allowed us to account for multiple confounding factors, including sociodemographic characteristics, residential features, and the baseline mental health status. Finally, we investigated the interactive and synergistic effects of housing and neighborhood quality on urban mental health, which has seldom been investigated in previous studies.

However, we acknowledge certain limitations in this study. One limitation is the relatively low response rate (51%), which may have resulted in selection bias. Yet, our response rate was comparable to previous population-based surveys [34]. Our study is also similar to other observational studies that are susceptible to residual confounding by unobservable variables or reverse causality bias (i.e., a pre-existing psychological disturbance leads to a negative perception of the environment). Furthermore, it is important to note that the assessment of housing and neighborhood qualities in this study relied on participants’ subjective evaluations. Subjective measures capture the genuine effects of individuals’ environmental attributes, considering their perceptions and prior knowledge of the living environment. Subjective measures also demonstrated good psychometric properties and reliabilities with objective measures of the environment [16]. Lastly, participants’ perceptions of their living environment over the past 3 years were gathered during the follow-up interview. As such, no information regarding the change in environmental perception, for example, during or after the COVID-19 pandemic was collected [35], and therefore, the trajectory of environmental risk factors on mental health should be further examined.

## Conclusion

This study has significant implications for the impact of the living environment on mental health in the general population of Hong Kong. Our study demonstrated that perception of living environment significantly affected the risk of developing CMD over time. Individual factors such as age and socioeconomic status play crucial roles in the perceived environmental qualities and might have acted as moderators between living environment and mental health. Future studies should evaluate the effects of specific characteristics of environmental qualities on mental health. Our findings also highlighted the synergistic effects of housing and neighborhood quality on mental health. The effects of the neighborhood on mental health were especially dominant in participants with mediocre housing quality. These findings further confirm the prominent role of neighborhood quality in urban mental health. All in all, the findings of this study will shed light on the ecological perspective of mental health and facilitate future urban planning to build a better neighborhood in Hong Kong.

### Supplementary Information

ESM 1 (DOCX 28 kb)
