# Supplementary material for: Individual and Interactive Effects of Housing and Neighborhood Quality on Mental Health in Hong Kong: A Retrospective Cohort Study
Source: J Urban Health. 2024 May 8;101(4):804–14. doi: 10.1007/s11524-024-00869-5 (PMC11329467; doi:10.1007/s11524-024-00869-5)
Supplement: Supplementary file 1 — (DOCX 28 kb) [file 11524_2024_869_MOESM1_ESM.docx]

| Supplementary Table 1 Housing quality by sociodemographic and residential characteristics (N=962) | | | |
| --- | --- | --- | --- |
|  | **Mean (SD)** | **Sig.**^a^ | **Pairwise comparison^b^** |
| Baseline age |  | 0.011 | Age 16–30 vs. Age 61–75, p=0.012 |
| 16–30 | 33.68 (5.78) |  |  |
| 31–45 | 34.96 (5.98) |  |  |
| 46–60 | 34.48 (6.29) |  |  |
| 61–75 | 35.84 (5.85) |  |  |
| Sex |  | 0.564 | -- |
| Male | 34.92 (5.80) |  |  |
| Female | 34.76 (6.17) |  |  |
| Education level |  | 0.005 | Upper vs. Post-secondary, p=0.003  Lower vs. Post-secondary, p=0.050  No schooling/primary vs. Post-secondary, p=0.275 |
| No schooling/primary | 35.06 (5.97) |  |  |
| Lower secondary | 34.53 (6.55) |  |  |
| Upper secondary | 34.26 (6.05) |  |  |
| Post-secondary | 36.04 (5.50) |  |  |
| Marital status |  | 0.009 | Never married vs. Married/cohabit, p=0.011 |
| Married/cohabit | 35.35 (6.15) |  |  |
| Never married | 33.88 (5.57) |  |  |
| Divorced/separated/widowed | 34.43 (6.22) |  |  |
| Employment status |  | <0.001 | Unemployed/not working vs. Working, p=0.050  Unemployed/not working vs. Retired, p=0.002  Unemployed/not working vs. Housewife, p=0.003 |
| Employed | 34.65 (6.03) |  |  |
| Retired | 35.92 (5.92) |  |  |
| Housewife | 35.72 (6.47) |  |  |
| Student | 33.79 (5.10) |  |  |
| Unemployed/not working | 32.71 (5.82) |  |  |
| Monthly household income (HKD) |  | <0.001 | <15000 vs. ≥60000, p<0.001  15000–24999 vs. ≥60000, p<0.001 |
| <15000 | 34.09 (5.97) |  |  |
| 15000–24999 | 33.77 (6.22) |  |  |
| 25000–39999 | 35.77 (6.01) |  |  |
| 40000–59999 | 35.66 (5.79) |  |  |
| ≥60000 | 37.34 (5.08) |  |  |
| Housing type |  | <0.001**6.29***** | -- |
| Public housing | 33.58 (5.74) |  |  |
| Private housing | 35.91 (6.12) |  |  |
| Housing size (square feet, in quintile) |  | <0.001**6** | ≤20% vs. 41-60%, p=0.008  ≤20% vs. 61-80%, p<0.001  ≤20% vs. >80%, p<0.001  21-40% vs. 61-80%, p=0.015  21-40% vs. >80%, p<0.001  41-60% vs. >80%, p<0.001 |
| ≤20% | 32.68 (6.19) |  |  |
| 21-40% | 34.20 (5.86) |  |  |
| 41-60% | 34.70 (6.11) |  |  |
| 61-80% | 36.09 (5.49) |  |  |
| >80% | 37.41 (5.40) |  |  |
| Housing tenure status |  | <0.001**6** | -- |
| Owner | 36.47 (5.78) |  |  |
| Renter | 33.34 (6.16) |  |  |
| Year of property age |  | 0.437 | -- |
| 3–10 | 35.79 (5.05) |  |  |
| 11–20 | 34.93 (5.60) |  |  |
| 21–30 | 35.03 (5.74) |  |  |
| 31–40 | 34.70 (6.50) |  |  |
| >40 | 33.67 (7.47) |  |  |
| SD, standard deviation; HKD, Hong Kong Dollar. | | | |
| ^a^Kruskal-Wallis test with test statistics. | | | |
| ^b^Pairwise comparisons adjusted by the Bonferroni correction for multiple tests. | | | |

| Supplementary Table 2 Neighborhood quality by sociodemographic and residential characteristics (N=962) | | | |
| --- | --- | --- | --- |
|  | **Mean (SD)** | **Sig.**^a^ | **Pairwise comparison^b^** |
| Baseline age |  | 0.002 | Age 16–30 vs. Age 31–45, p=0.029  Age 16–30 vs. Age 61–75, p=0.005 |
| 16–30 | 33.68 (5.78) |  |  |
| 31–45 | 34.96 (5.98) |  |  |
| 46–60 | 34.48 (6.29) |  |  |
| 61–75 | 35.84 (5.85) |  |  |
| Sex |  | 0.072 | -- |
| Male | 35.75 (5.33) |  |  |
| Female | 35.19 (5.49) |  |  |
| Education level |  | 0.004 | Upper vs. Post-secondary, p=0.003 |
| No schooling/primary | 35.81 (5.61) |  |  |
| Lower secondary | 35.44 (5.65) |  |  |
| Upper secondary | 34.68 (5.34) |  |  |
| Post-secondary | 36.33 (5.15) |  |  |
| Marital status |  | <0.001 | Never married vs. Married/cohabit, p<0.001 |
| Married/cohabit | 35.99 (5.51) |  |  |
| Never married | 34.15 (4.87) |  |  |
| Divorced/separated/widowed | 35.07 (5.69) |  |  |
| Employment status |  | 0.005 | Unemployed/not working vs. Retired, p=0.015  Unemployed/not working vs. Housewife, p=0.039 |
| Employed | 35.25 (5.27) |  |  |
| Retired | 36.31 (5.62) |  |  |
| Housewife | 36.10 (5.73) |  |  |
| Student | 34.00 (5.15) |  |  |
| Unemployed/not working | 33.73 (5.46) |  |  |
| Monthly household income (HKD) |  | <0.001 | <15000 vs. ≥60000, p=0.019  15000–24999 vs. 40000–59999, p=0.039  15000–24999 vs. ≥60000, p<0.001 |
| <15000 | 35.08 (5.37) |  |  |
| 15000–24999 | 34.32 (5.26) |  |  |
| 25000–39999 | 35.85 (6.05) |  |  |
| 40000–59999 | 36.25 (5.34) |  |  |
| ≥60000 | 36.98 (4.80) |  |  |
| Housing type |  | <0.001**6.29***** | -- |
| Public housing | 34.66 (5.16) |  |  |
| Private housing | 35.97 (5.63) |  |  |
| Housing size (square feet, in quintile) |  | 0.016 | ≤20% vs. >80%, p=0.050 |
| ≤20% | 34.56 (5.41) |  |  |
| 21-40% | 34.92 (5.53) |  |  |
| 41-60% | 35.74 (5.46) |  |  |
| 61-80% | 35.85 (5.04) |  |  |
| >80% | 36.22 (5.60) |  |  |
| Housing tenure status |  | <0.001**6** | -- |
| Owner | 35.96 (5.47) |  |  |
| Renter | 34.60 (5.33) |  |  |
| Year of property age |  | 0.179 | -- |
| 3–10 | 34.83 (5.34) |  |  |
| 11–20 | 35.04 (5.38) |  |  |
| 21–30 | 35.34 (5.20) |  |  |
| 31–40 | 36.20 (5.54) |  |  |
| >40 | 34.98 (6.08) |  |  |
| SD, standard deviation; HKD, Hong Kong Dollar. | | | |
| ^a^Kruskal-Wallis test with test statistics. | | | |
| ^b^Pairwise comparisons adjusted by the Bonferroni correction for multiple tests. | | | |

| **Supplementary Table 3** Association between sociodemographic and residential characteristics, housing quality and CMD at follow-up (N=962)^a^ | | | | |
| --- | --- | --- | --- | --- |
|  | **B** | **SE** | **Wald statistic** | **aOR (95% CI)** |
| **Step 1** |  |  |  |  |
| Sex (female) | 0.20 | 0.27 | 0.56 | 1.23 (0.72-2.08) |
| Baseline age |  |  |  |  |
| 16–30 | Reference |  |  |  |
| 31–45 | 0.15 | 0.44 | 0.12 | 1.17 (0.50-2.75) |
| 46–60 | -0.12 | 0.45 | 0.07 | 0.89 (0.37-2.16) |
| 61–75 | -0.63 | 0.61 | 1.09 | 0.53 (0.16-1.74) |
| Marital status |  |  |  |  |
| Married/cohabit | Reference |  |  |  |
| Never married | 0.00 | 0.35 | 0.00 | 1.00 (0.51-1.99) |
| Divorced/separated/widowed | 0.09 | 0.31 | 0.08 | 1.09 (0.60-1.99) |
| Education level |  |  |  |  |
| No schooling/primary | Reference |  |  |  |
| Lower secondary | 0.74 | 0.37 | 3.97 | 2.10 (1.01-4.37)^*^ |
| Upper secondary | -0.31 | 0.37 | 0.68 | 0.74 (0.35-1.53) |
| Post-secondary | -0.40 | 0.55 | 0.54 | 0.67 (0.23-1.96) |
| Monthly household income (HKD) |  |  |  |  |
| <15000 | Reference |  |  |  |
| 15000–24999 | -0.50 | 0.35 | 2.07 | 0.61 (0.31-1.20) |
| 25000–39999 | -0.67 | 0.41 | 2.67 | 0.51 (0.23-1.14) |
| 40000–59999 | -0.50 | 0.48 | 1.09 | 0.60 (0.24-1.56) |
| ≥60000 | -0.40 | 0.54 | 0.54 | 0.67 (0.23-1.93) |
| Employment status |  |  |  |  |
| Employed | Reference |  |  |  |
| Retired | -0.64 | 0.49 | 1.69 | 0.53 (0.20-1.38) |
| Housewife | 0.19 | 0.35 | 0.30 | 1.21 (0.61-2.42) |
| Student | -1.10 | 0.74 | 2.18 | 0.34 (0.08-1.43) |
| Unemployed/not working | 0.69 | 0.39 | 3.11 | 1.99 (0.93-4.26) |
| **Step 2** |  |  |  |  |
| Public housing | -0.80 | 0.40 | 4.03 | 0.45 (0.21-0.98)^*^ |
| Tenured housing | -0.95 | 0.37 | 6.56 | 0.39 (0.19-0.80)^*^ |
| Year of property age |  |  |  |  |
| 3–10 | Reference |  |  |  |
| 11–20 | 0.49 | 0.61 | 0.62 | 1.62 (0.49-5.41) |
| 21–30 | 0.85 | 0.62 | 1.88 | 2.33 (0.70-7.80) |
| 31–40 | 0.47 | 0.64 | 0.55 | 1.60 (0.46-5.55) |
| >40 | 0.06 | 0.71 | 0.01 | 1.06 (0.27-4.26) |
| **Step 3** |  |  |  |  |
| CMD at baseline | 2.27 | 0.24 | 88.27 | 9.67 (6.02-15.52)^***^ |
| **Step 4** |  |  |  |  |
| Quality of housing | -0.05 | 0.02 | 7.28 | 0.95 (0.91-0.98)^**^ |
| B, coefficient on the variable; CI, confidence interval; CMD, common mental disorder; HKD, Hong Kong Dollar; aOR, adjusted odds ratio; SE, standard error.  ^a^Final stepwise logistic regression model: χ^2^=194.09, df=25, p<0.001; Nagelkerke R^2^=0.390.  ^***^p<0.001; ^**^p<0.01; ^*^p<0.05. | | | | |

| Supplementary Table 4 Association between housing and neighborhood quality (N=962)^a^ | | | | | | | | | | | | |
| --- | --- | --- | --- | --- | --- | --- | --- | --- | --- | --- | --- | --- |
|  | | Neighborhood quality | | | | | | | | | | |
|  |  | Lighting | Air quality | Hygiene | Serenity | Transportation | Shopping | Catering | Medical facilities | Park and recreation | Security | Total |
| **Housing quality** | Lighting | 0.475 | 0.366 | 0.349 | 0.372 | 0.264 | 0.213 | 0.165 | 0.227 | 0.338 | 0.370 | 0.440 |
|  | Air quality | 0.461 | **0.585** | 0.486 | 0.496 | 0.238 | 0.192 | 0.182 | 0.221 | 0.356 | 0.360 | **0.501** |
|  | Hygiene | 0.436 | 0.420 | 0.459 | 0.436 | 0.317 | 0.280 | 0.267 | 0.235 | 0.337 | 0.412 | **0.500** |
|  | Serenity | 0.455 | 0.457 | 0.475 | **0.575** | 0.333 | 0.288 | 0.241 | 0.333 | 0.357 | 0.404 | **0.545** |
|  | Spacing | 0.393 | 0.377 | 0.398 | 0.422 | 0.295 | 0.307 | 0.278 | 0.307 | 0.430 | 0.392 | **0.507** |
|  | Kitchen facilities | 0.441 | 0.370 | 0.397 | 0.371 | 0.324 | 0.260 | 0.243 | 0.262 | 0.370 | 0.377 | 0.470 |
|  | Toilet facilities | 0.438 | 0.404 | 0.472 | 0.415 | 0.294 | 0.281 | 0.249 | 0.312 | 0.436 | 0.441 | **0.524** |
|  | Furnishings | 0.347 | 0.366 | 0.409 | 0.412 | 0.290 | 0.301 | 0.253 | 0.339 | 0.396 | 0.347 | 0.492 |
|  | Architecture and design | 0.396 | 0.426 | 0.426 | 0.444 | 0.237 | 0.254 | 0.213 | 0.326 | 0.437 | 0.393 | **0.504** |
|  | Structure safety | **0.517** | 0.442 | 0.494 | 0.434 | 0.317 | 0.319 | 0.251 | 0.332 | 0.457 | **0****.527** | **0.573** |
|  | Total | **0.565** | **0.560** | **0.582** | **0.584** | 0.382 | 0.356 | 0.303 | 0.389 | **0.518** | **0.533** | **0.676** |
| ^a^Spearman’s rank correlation analysis with correlation coefficient (*r*), all associations remained significant after Bonferroni correction for multiple testing (p<0.0005). *r*>0.5 are indicated in boldface type. | | | | | | | | | | | | |

| **Supplementary Table 5** Association between sociodemographic and residential variables, housing and neighborhood quality (with interaction term) and CMD at follow-up (N=962)^a,b^ | | | | |
| --- | --- | --- | --- | --- |
|  | **B** | **SE** | **Wald statistic** | **aOR (95% CI)** |
| **Step 1** |  |  |  |  |
| Sex, Baseline age, Marital status, Education level, Monthly household income, Employment status | | | | |
| **Step 2** |  |  |  |  |
| Public housing, Tenured housing, Year of property age | | | | |
| **Step 3** |  |  |  |  |
| Baseline CMD | | | | |
| **Step 4** |  |  |  |  |
| Quality of housing | 0.04 | 0.10 | 0.12 | 1.04 (0.85-1.27) |
| Quality of neighborhood | -0.04 | 0.10 | 0.14 | 0.96 (0.79-1.18) |
| Quality of housing x neighborhood^c^ | 0.00 | 0.00 | 0.20 | 1.00 (0.99-1.00) |
| B, coefficient on the variable; CI, confidence interval; CMD, common mental disorder; aOR, adjusted odds ratio; SE, standard error.  ^a^Final stepwise logistic regression model: χ^2^=202.59, df=27, p<0.001; Nagelkerke R^2^=0.405.  ^b^Housing and neighborhood quality are the variables of interest, details of Step 1-3 are not shown.  ^c^Interaction term of housing quality and neighborhood quality. | | | | |
